# Association between vitamin D deficiency and health-related quality of life in patients with chronic kidney disease from the KNOW-CKD study

**DOI:** 10.1371/journal.pone.0174282

**Published:** 2017-04-27

**Authors:** Tae Ryom Oh, Chang Seong Kim, Eun Hui Bae, Seong Kwon Ma, Seung Hyeok Han, Su-Ah Sung, Kyubeck Lee, Kook Hwan Oh, Curie Ahn, Soo Wan Kim, Representatives of the KNOW-CKD Investigator Group

**Affiliations:** 1 Department of Internal Medicine, Chonnam National University Medical School, Gwangju, Korea; 2 Depatment of Internal Medicine, Yonsei University College of Medicine, Seoul, Korea; 3 Department of Internal Medicine, Eulji University, Seoul, Korea; 4 Department of Internal Medicine, Kangbuk Samsung Hospital, Sungkyunkwan University, Seoul, Korea; 5 Department of Internal Medicine, Seoul National University, Seoul, Korea; University of Alabama at Birmingham, UNITED STATES

## Abstract

Vitamin D deficiency is a growing health problem in both the general population and in patients with chronic kidney disease (CKD). However, the relationship between serum 25-hydroxyvitamin D levels and health-related quality of life in CKD is not well established. This study examined the association between vitamin D deficiency and quality of life in pre-dialysis CKD patients. Serum 25-hydroxyvitamin D levels and the Korean version of the Kidney Disease Quality of Life short form were obtained for 1844 pre-dialysis CKD patients in the prospective KoreaN cohort Study for Outcomes in patients With Chronic Kidney Disease (KNOW-CKD). The baseline estimated glomerular filtration rate was 50.26 ± 0.71 mL/min/1.73 m^2^. We identified 1294 (70.2%) patients with vitamin D deficiency, defined as a 25-hydroxyvitamin D level < 20 ng/ml. The scores of the kidney disease component summary, physical component summary, and mental component summary in the vitamin D deficiency group were significantly lower compared to the scores of those without vitamin D deficiency. The serum 25-hydroxyvitamin D level was independently associated with the kidney disease component summary and mental component summary scores (*β* = 0.147, *p* = 0.003 and *β* = 0.151, *p* = 0.047). In conclusion, there was a significant association between serum 25-hydroxyvitamin D levels and kidney disease component summary and mental component summary scores in pre-dialysis CKD patients.

## Introduction

Chronic kidney disease (CKD) is a major public health concern with rising prevalence. With advances in medicine, the life expectancy of CKD patients has been increasing, and importantly, this has been paralleled by an improvement in patients’ quality of life (QOL). QOL refers to the general well-being of individuals, and covers a wide range of contexts including healthcare, employment, and politics, among others. Health-Related Quality of Life (HRQOL) is a related concept that focuses on the effects of illness, and specifically on the impact that treatment may have on QOL. It can help us make out the distinction between aspects of life related to health. A number of definitions have been proposed to explain the concept of HRQOL, and it has now been generally accepted that HRQOL represents the positive and negative aspects of patients’ symptoms, including emotional, social, cognitive functions, disease burden, and treatment side effects [1].

Vitamin D deficiency is a growing health problem in both the general population and in patients with CKD [2, 3]. It is known to cause several problems, including depression, muscle ache and weakness, osteoporosis, periodontitis, and rickets, among others [4, 5] Some observational studies have identified an important relationship between vitamin D deficiency and decreased glomerular filtration rate (GFR) in patients with CKD [6, 7]. The crucial role of vitamin D in CKD extends beyond the classic effects on calcium and phosphorous homeostasis, and includes potential effects on extra-mineral metabolism, including the regulation of the immune system and of kidney function. Some studies have shown that vitamin D deficiency in hemodialysis patients results in the loss of muscle mass and strength [8, 9]. In addition, patients with vitamin D deficiency had lower self-reported levels of physical activity and HRQOL in end stage renal disease (ESRD) [10]. Interestingly, vitamin D plays an important role in the regulation of immune function [11, 12], and its deficiency was related with an increased rate of infection. In fact, both infection and CKD-Mineral and Bone Disorders (MBD) affect the survival and hospitalization rates of patients with CKD, and vitamin D underlies both of these. Taken together, the studies described above suggest that vitamin D may affect HRQOL. However, this relationship has not been formally investigated in pre-dialysis patients. In this study, we analyzed the correlation between vitamin D deficiency and HRQOL in CKD patients.

## Methods

### Study population and ethic statement

We reviewed baseline data from the KoreaN Cohort Study for Outcome in Patients With Chronic Kidney Disease (KNOW-CKD), a nationwide prospective cohort study which included non-dialysis patients with stage 1–5 CKD. KNOW-CKD was launched when the research contract between "the Korea Centers for Disease Control and Prevention" and "Seoul National University Hospital" was established in Feb, 2011. The study protocol was approved by the ethical committee of each participating center. Each center started to enroll the patients after getting the ethical approvals of its own. The first patient was enrolled on June 30th, 2011 by Seoul National University Hospital (SNUH) after getting the ethical approval in May 2011 at SNUH. A total of 2238 patients were enrolled from June, 2011 to January, 2016. Data were collected by a well-trained study coordinator using a standardized case report form and protocol. The detailed design and methods of the KNOW-CKD study have been previously published [13]. All procedures performed in human participants were in accordance with the ethical standards of the institutional and national research committee at which the studies were conducted (IRB approval number CNUH-2011-092), and with the 1964 Helsinki declaration and its later amendments or comparable ethical standards. The study protocol was approved by the institutional review board at each participating clinical center. All participating patients provided written informed consent. For our analysis, we obtained information on serum vitamin D levels and quality of life for 1844 non-dialysis patients with CKD.

### Data collection and survey instruments

CKD stages are defined as GFR > 90 (CKD stage 1); 60 ≤ GFR < 90 (CKD stage 2); 30 ≤ GFR < 60 (CKD stage 3); 15 ≤ GFR < 30 (CKD stage 4); and GFR < 15 mL/min/1.73 m^2^ (CKD stage 5). The eGFR was calculated by using the Modification of Diet in Renal Disease (MDRD) study equation [14]. Age, gender, eGFR, body mass index (BMI), work status, hemoglobin, nutritional factors (albumin and lipid profile), presence of metabolic syndrome, high sensitive C-reactive protein (hsCRP), serum 25-hydroxyvitamin D (25(OH)D), which is a surrogate marker for the vitamin D status of the body, serum parathyroid hormone (PTH), diabetes mellitus (DM), hypertension (HTN), education level and economic status were assessed in all patients. Economic status was classified by monthly income as ‘Low’ (< ₩ 1,500,000), ‘Mid’ (₩ 1,500,000 ~ 4,500,000), or ‘High’ (≥₩ 4,500,000). Patients were divided into a control (vitamin D replete) and vitamin D deficiency group (serum 25(OH)D < 20 ng/ml). The Korean version 1.3 of Kidney Disease Quality of Life short form (KDQOL-SF) was used to evaluate HRQOL [15]. The KDQOL-SF is composed of a kidney disease component summary (KDCS), a physical component summary (PCS), and a mental component summary (MCS). The KDCS includes 43 kidney-disease targeted items, while the PCS and MCS each include a generic 36-item health survey. The KDCS includes eleven subscales: (1) Symptom/problem, (2) Effects of kidney disease, (3) Burden of kidney disease, (4) Work status, (5) Cognitive function, (6) Quality of social interaction, (7) Sexual function, (8) Sleep, (9) Social support, (10) Staff encouragement, and(11) Patients satisfaction. The PCS and MCS contains each four subscales: (1) Physical Function, (2) Role Physical limitation due to physical problems, (3) Bodily Pain, (4) General Health, (5) Vitality, (6) Role-Emotional, and(7) Social Function, (8) Mental Health. The former four subscales are summarized in a Physical Component Summary (PCS) and the later four subscales are summarized in a Mental Component Summary (MCS). Answers to each question were converted into SF-36 equivalent scores, and each scale ranges from 0 to 100.

### Statistical analysis

Data were analyzed using SPSS 20 for Windows (SPSS Inc., Chicago, IL, USA). We used frequency analysis to evaluate the prevalence of vitamin D deficiency. A chi-square test for categorical variables and a student t-test for continuous variables were used to survey the differences and to compare HRQOL scores between the two groups. We also used linear regression analysis to define variables related with each component summary score of KDQOL-SF. Then, stepwise multivariable linear regression analyses were performed to identify the independent risk factors associated with HRQOL. The only verified variables, which had statistical significance in univariate analysis, were used in multiple regression analysis. The following variables required adjustment: (1) KDCS and MCS; age, sex, eGFR, work status, diabetes mellitus, level of education, economic status, serum 25(OH)D, parathyroid hormone, hemoglobin, albumin, HDL-C (high density lipid-cholesterol) and hsCRP (2) PCS; age, sex, eGFR, work status, diabetes mellitus, level of education, economic status, serum 25(OH)D, parathyroid hormone, hemoglobin, albumin, HDL-C (high density lipid-cholesterol), hsCRP and waist circumference. Results are presented as mean ± standard error and *P* < 0.05 was considered statistically significant.

## Results

### Clinical characteristics

Table 1 shows the baseline demographic characteristics of the study participants. A total of 1844 patients were included in the study, and their mean age was 50 years old. The majority of patients (1135, 61.6%) were male, and 612 patients (33.2%) had DM. Approximately one half (55.6%) of subjects were employed, and 42.5% had at least college-level education. The mean serum creatinine level was 1.83±0.03 mg/dl, and the mean eGFR calculated by the MDRD equation was 50.26±0.71 ml/min/1.73m^2^.

**Table 1:** Baseline clinical characteristics of patients.

	Overall (n = 1844)	Control (n = 550)	Vitamin D deficiency (n = 1294)	*P* value
Age (years)	50.3±0.71	55.3±0.51	52.8±0.34	0.000
Male (%)	1135 (61.6)	160 (29.1)	549 (42.4)	0.000
Waist circumference (cm)	87.3±0.23	87.0±0.39	87.5±0.28	0.279
Work status (%)	1025 (55.6)	325 (59.1)	700 (54.1)	0.048
Diabetes mellitus (%)	612 (33.2)	162 (29.5)	450 (34.9)	0.026
Hypertension (%)	1747 (94.7)	518 (94.2)	1229 (95.0)	0.484
Education^a^				
Elementary school	180 (9.8)	51 (9.4)	129 (10.1)	
Middle school	216 (11.7)	63 (11.6)	153 (12.0)	
High school	642 (34.8)	194 (35.7)	448 (35.1)	
University	783 (42.5)	236 (43.4)	547 (42.8)	
Economic status				
Low^b^	443 (24.7)	150 (27.7)	293 (23.4)	
Mid	924 (51.6)	282 (52.1)	642 (51.3)	
High^b^	425 (23.7)	109 (20.1)	316 (25.3)	
Metabolic syndrome	1093 (59.3)	305 (27.9)	788 (72.1)	0.030
Blood urea nitrogen (mg/dl)	28.2±0.37	27.7±0.61	28.5±0.45	0.320
Creatinine (mg/dl)	1.8±0.03	1.7±0.04	1.9±0.03	0.007
eGFR (ml/min/1.73m^2^)	50.3±0.71	50.5±1.21	50.2±0.86	0.820
Serum 25(OH)D (ng/dl)	17.7±0.17	26.5±0.29	14.0±0.10	0.000
Parathyroid hormone	70.1±2.64	54.5±2.66	78.2±3.71	0.000
Uncorrected calcium (mg/dl)	9.07±0.02	9.17±0.03	9.04±0.02	0.001
Hemoglobin (g/dl)	12.7±0.05	13.0±0.10	12.6±0.06	0.001
Albumin (g/dl)	4.2±0.11	4.2±0.02	4.11±0.01	0.000
Total cholesterol (mg/dl)	173.3±0.92	169.1±1.43	175.1±1.16	0.001
LDL-C (mg/dl)	95.3±0.76	93.1±1.21	96.3±0.95	0.038
HDL-C (mg/dl)	49.2±0.36	49.0±0.61	49.3±0.44	0.704
Triglyceride (mg/dl)	156.9±2.26	140.9±3.68	163.5±2.79	0.000
C-reactive protein (mg/l)	1.9±0.12	1.8±0.21	1.9±0.14	0.460

^a^ No statistically significant difference between two groups

^b^ *P* < 0.048 by Chi-square test with Bonferroni's correction

Abbreviations: eGFR, estimated glomerular filtration rate; LDL-C, low density lipoprotein cholesterol; HDL-C, high density lipoprotein cholesterol

There were 550 patients in the control group and 1294 patients in the vitamin D deficiency group. In both groups, the proportion of men was higher than that of women. The level of serum 25(OH)D was 26.51±0.29 ng/ml in the control group and 13.97±0.10 ng/ml in the vitamin D deficiency group. The mean age of the control and vitamin D deficiency groups was 55 and 52 years old, respectively. The prevalence of DM in the vitamin D deficiency group was higher than in the control group (29.5% vs. 34.9%). The eGFR was 50.50±1.21 ml/min/1.73m^2^ in the control group, and 50.16±0.86 ml/min/1.73m^2^ in the vitamin D deficiency group. The level of education did not show any difference between the two groups. There was a significant difference between groups with respect to the number of participants in the low and high economic status categories (p < 0.048 by Chi-square test with Bonferroni's correction). There were 325 employed patients (59.1%) in the control group and 700 employed patients (54.1%) in the vitamin D deficiency group. The hsCRP and high density lipid levels did not differ between the two groups.

### Association between HRQOL and vitamin D levels in CKD patients

Each component summary of HRQOL {PCS, MCS, KDCS)} and subscales were evaluated and compared between control and vitamin D deficiency groups (Table 2). Among disease-specific KDCS domains, vitamin D deficient patients showed significantly lower scores in the symptom/problem, effects of kidney disease, burden of kidney disease, work status, cognitive function, social support and patients’ satisfaction domains (p < 0.05). There was no statistically significant difference between groups in scores for quality of social interaction, sexual function, sleep, and staff encouragement. Among SF-36 domains, the vitamin D deficient patients showed significantly lower scores in physical function, role physical, pain, emotional well-being, social function and energy/fatigue domains (p < 0.05). The mean values for KDCS, PCS and MCS in the vitamin D deficient group were lower compared to those in the control group (74.74±0.55 vs. 71.48±0.36, 74.93±0.76 vs. 71.38±0.52 and 71.41±0.77 vs. 68.89±0.51, respectively, p < 0.05) (Table 2).

**Table 2:** Comparison of KDQOL-SF^™^ scores between vitamin D deficiency and control groups.

	Vitamin D sufficiency	Vitamin D deficiency	*P* value
Kidney disease component summary	74.74±0.55	71.48±0.36	0.000
Symptom/problem	87.45±0.53	85.29±0.40	0.001
Effects of kidney disease	66.82±1.61	59.18±1.11	0.000
Burden of kidney disease	49.93±1.91	42.97±1.25	0.002
Work status	83.63±0.83	81.56±0.55	0.038
Cognitive function	88.77±0.59	86.81±0.41	0.006
Social interaction	89.82±0.55	88.72±0.40	0.118
Sexual function	73.27±0.71	71.75±0.48	0.080
Sleep	64.01±0.99	61.14±0.62	0.104
Social support	68.95±1.14	64.84±0.76	0.003
Staff encouragement	75.94±0.74	74.32±0.47	0.061
Patients’ satisfaction	73.42±0.96	69.73±0.67	0.002
Physical component summary	74.93±0.76	71.38±0.52	0.000
Physical functioning	86.99±0.68	84.55±0.49	0.004
Role-physical	83.35±1.31	78.23±0.96	0.002
Pain	86.12±0.84	81.36±0.62	0.000
General health perceptions	42.77±0.96	40.89±0.59	0.097
Mental component summary	71.41±0.77	68.89±0.51	0.007
Emotional well-being	67.73±0.77	62.58±0.48	0.016
Role-emotional	82.19±1.46	79.12±1.01	0.085
Social function	86.56±0.82	84.27±0.58	0.022
Energy/fatigue	51.49±0.84	49.17±0.52	0.015

Abbreviations: KDQOL-SF, Korean version 1.3 of Kidney Disease Quality of Life short form

### Vitamin D deficiency as an independent risk factor for impaired HRQOL

We surveyed the relationship between clinical variables and each component summary of KDQOL-SF: KDCS, PCS and MCS (Tables 3–5). We performed univariate and stepwise multiple linear regression analysis with factors which were related to quality of life including sex, age, work status, co-morbidity (DM and HTN), renal function, serum 25(OH)D level, hemoglobin, serum albumin, hsCRP, lipid profile (to reflect nutritional status), markers for CKD-MBD, education level, economic status and some other variables.

**Table 3:** The results of regression model to explain variables related with kidney disease component summary (KDCS) scores.

KDCS	Unadjusted		Multivariable adjusted ^a^	
	*β*±SE	*P* value	*β*±SE	*P* value
Age	−0.165±0.025	0.000	−	0.097
Sex (male)	4.827±0.617	0.000	2.287±0.931	0.014
eGFR (ml/min/1.73m^2^)	0.109±0.010	0.000	0.081±0.015	0.000
Unemployed status	−10.235±0.566	0.000	−7.421±0.901	0.000
Waist circumference (cm)	−0.043±0.031	0.176	−	−
Diabetes mellitus (%)	−6.735±0.629	0.000	−4.500±0.909	0.000
Hypertension (%)	−1.155±1.367	0.398	−	−
Education				
Elementary school	−10.172±1.053	0.000	−5.050±1.537	0.001
Middle school	−6.101±0.979	0.000	−2.771±1.362	0.042
High school	−1.934±0.678	0.004	−2.516±0.978	0.010
University	Reference category	−	−	−
Economic status				
Low	Reference category	−	−	−
Mid	6.720±0.740	0.000	−	0.170
High	9.847±0.857	0.000	−	0.548
Serum 25(OH)D (ng/dl)	0.265±0.040	0.000	0.147±0.049	0.003
Parathyroid hormone	−0.043±0.007	0.000	−	0.056
Hemoglobin (g/dl)	1.503±0.126	0.000	−	0.080
Albumin (g/dl)	4.246±0.618	0.000	2.891±0.910	0.002
Total cholesterol (mg/dl)	0.001±0.008	0.899	−	−
LDL-C (mg/dl)	0.005±0.009	0.627	−	−
HDL-C (mg/dl)	0.048±0.020	0.017	−	0.513
Triglyceride (mg/dl)	−0.005±0.003	0.103	−	−
hsCRP (mg/l)	−0.188±0.061	0.002	−	0.192

^a^ Stepwise multiple regression adjusted for factors including age, sex (male), eGFR, unemployed status, diabetes mellitus, education, economic status, serum 25(OH)D, parathyroid hormone, hemoglobin, albumin, HDL-C and hsCRP.

Abbreviations: SE, standard error; eGFR, estimated glomerular filtration rate; LDL-C, low density lipoprotein cholesterol; HDL-C, high density lipoprotein cholesterol; hsCRP, high-sensitive C-reactive protein

**Table 4:** The results of regression model to explain variables related with physical component summary (PCS) scores.

PCS	Unadjusted		Multivariable adjusted ^a^	
	*β*±SE	p-value	*β*±SE	p-value
Age	−0.326±0.035	0.000	−	0.841
Sex (male)	7.502±0.871	0.000	4.388±1.376	0.001
eGFR (ml/min/1.73m^2^)	0.144±0.014	0.000	0.069±0.022	0.002
Unemployed status	−11.641±0.826	0.000	−8.097±1.335	0.000
Waist circumference (cm)	−0.161±0.044	0.000	−	0.120
Diabetes mellitus (%)	−9.960±0.889	0.000	−8.071±1.340	0.000
Hypertension (%)	−2.037±1.935	0.293	−	−
Education				
Elementary school	−18.228±1.442	0.000	−11.399±2.280	0.000
Middle school	−11.654±1.341	0.000	−4.505±2.018	0.026
High school	−4.435±0.929	0.000	−5.061±1.450	0.001
University	Reference category	−	−	−
Economic status				
Low	Reference category	−	−	−
Mid	11.034±1.035	0.000	−	0.446
High	15.289±1.199	0.000	−	0.065
Serum 25(OH)D (ng/dl)	0.308±0.057	0.000	−	0.075
Parathyroid hormone	−0.042±0.010	0.000	−	0.524
Hemoglobin (g/dl)	2.063±0.179	0.000	−	0.536
Albumin (g/dl)	5.410±0.878	0.000	3.705±1.344	0.006
Total cholesterol (mg/dl)	−0.008±0.011	0.475	−	−
LDL-C (mg/dl)	−0.003±0.013	0.835	−	−
HDL-C (mg/dl)	0.074±0.028	0.009	−	0.973
Triglyceride (mg/dl)	−0.008±0.005	0.074	−	−
hsCRP (mg/l)	−0.392±0.086	0.000	−0.360±0.146	0.014

^a^ Stepwise multiple regression adjusted for factors includin age, sex (male), eGFR, unemployed status, waist circumference, diabetes mellitus, education, economic status, serum 25(OH)D, parathyroid hormone, hemoglobin, albumin, HDL-C and hsCRP.

Abbreviations: SE, standard error; eGFR, estimated glomerular filtration rate; LDL-C, low density lipoprotein cholesterol; HDL-C, high density lipoprotein cholesterol; hsCRP, high-sensitive C-reactive protein

**Table 5:** The results of regression model to explain variables related with mental component summary (MCS) scores.

MCS	Unadjusted		Multivariable adjusted ^a^	
	*β*±SE	p-value	*β*±SE	p-value
Age	−0.158±0.035	0.000	0.141±0.064	0.027
Sex (male)	4.349±0.874	0.000	−	0.766
eGFR (ml/min/1.73m^2^)	0.102±0.014	0.000	0.072±0.024	0.002
Unemployed status	−7.803±0.842	0.000	−5.792±1.377	0.000
Waist circumference (cm)	−0.041±0.044	0.348	−	−
Diabetes mellitus (%)	−6.780±0.896	0.000	−4.887±1.420	0.001
Hypertension (%)	−1.563±1.916	0.415	−	−
Education				
Elementary school	−12.768±1.470	0.000	−10.101±2.403	0.000
Middle school	−8.161±1.367	0.000	−5.402±2.134	0.012
High school	−2.406±0.948	0.011	−4.609±1.512	0.002
University	Reference category	−	−	−
Economic status				
Low	Reference category	−	−	−
Mid	8.773±1.045	0.000	−	0.084
High	11.393±1.211	0.000	−	0.415
Serum 25(OH)D (ng/dl)	0.284±0.057	0.000	0.151±0.076	0.047
Parathyroid hormone	−0.033±0.009	0.001	−	0.903
Hemoglobin (g/dl)	1.504±0.180	0.000	−	0.373
Albumin (g/dl)	4.411±0.872	0.000	3.209±1.406	0.023
Total cholesterol (mg/dl)	−0.002±0.011	0.829	−	−
LDL-C (mg/dl)	0.002±0.013	0.882	−	−
HDL-C (mg/dl)	0.080±0.028	0.004	−	0.775
Triglyceride (mg/dl)	−0.005±0.005	0.300	−	−
hsCRP (mg/l)	−0.239±0.085	0.005	−	0.085

^a^ Stepwise multiple regression adjusted for factors included in age, sex (male), eGFR, unemployed status, diabetes mellitus, education, economic status, serum 25(OH)D, parathyroid hormone, hemoglobin, albumin, HDL-C and hsCRP.

Abbreviations: SE, standard error; eGFR, estimated glomerular filtration rate; LDL-C, low density lipoprotein cholesterol; HDL-C, high density lipoprotein cholesterol; hsCRP, high-sensitive C-reactive protein

The vitamin D deficiency group had lower scores in all HRQOL components. Work status, DM and the level of education were identified as important risk factors of all component summary scores. Also, serum albumin level significantly affected all composite summary scores. Economic status was only significant in univariate analysis. As expected, low income was related with lower HRQOL. Serum 25(OH)D level was an independent risk factor for KDCS & MCS (β = 0.147, p = 0.003 and β = 0.151, p = 0.047). There was no statistically significant relationship between serum 25(OH)D level and PCS in multiple regression analysis.

## Discussion

A quality of life assessment is critical to the holistic management of patients living with chronic diseases including CKD. Using data from the MDRD clinical trial, *Rocco et al*. [16] showed that decreased GFR in CKD patients was correlated with impaired quality of life assessed by the SF-36 health survey. Since then, many other studies have confirmed that physical function and viability affected patients’ quality of life, and that HRQOL was significantly reduced in ESRD patients when compared with the general population [17–19].

The KDQOL-SF consists of KDQOL instruments and generic SF-36 variables, and includes specific questions to assess symptom burden in dialysis patients. Although KDQOL-SF was originally developed to evaluate HRQOL in ESRD patients [20, 21], recent studies have validated the use of this questionnaire for pre-dialysis patients, and have revealed reduced QOL scores for pre-dialysis patients compared with the general population [22, 23]. There is also a paper showing that HRQOL is a powerful predictor of hospitalization and mortality. *Lowrie et al*. demonstrated that each 1-point increase in PCS was associated with a 2% drop in the relative risk of death and hospitalization, each 1-point increase in MCS was associated with a 2% drop in the relative risk of death and a 1% drop in the relative risk of hospitalization [24]. In this context, we might speculate that even though small differences of QOL were associated with hospitalization and mortality, and may predict patient outcomes.

In addition to a negative impact on HRQOL, it is well known that impaired GFR results in a decline in serum 25(OH)D levels in CKD patients. In this study, we showed that vitamin D deficient patients also have lower average HRQOL scores. The majority of HRQOL items which differed significantly between vitamin D deficient and control groups were KDCS, PCS and MCS. These items, which showed a difference between two groups, reflected the physical and mental burden of CKD, and were associated with physical or mental status such as working, daily life, well-being sense and mood.

Nevertheless, given that so many factors are associated with QOL (i.e. dietary habits, employment status, educational level, comorbid diseases, and a multitude of psychological variables), we performed multiple linear regression analysis with various variables, which were expected to relate with HRQOL, in order to verify the actual effect of serum 25(OH)D levels on HRQOL. In our study, The vitamin D deficiency group showed a lower score on all component summary scores. Regression analysis showed that serum 25(OH)D levels did not affect the PCS score, but did affect KDCS and MCS scores. It can be speculated that main component of PCS were associated with pain and activity, and the effect of other factors related to this part seems to be greater than that of vitamin D deficiency.

This study has some limitations worth noting. First, although the KNOW-CKD study is planned as a prospective observational study, these results are from an initial cross-sectional study. Therefore, well-designed, large randomized controlled trials are necessary to define whether vitamin D supplementation may improve HRQOL in CKD patients. Second, as mentioned, there is no universally accepted method to evaluate HRQOL in pre-dialysis patients, and thus a standardized method for measuring the HRQOL is needed. Third, all responses to the questionnaire were voluntary, and therefore we cannot exclude the possibility of selection bias. Finally, a novel pathway of vitamin D through CYP11A1 has been described which has not yet been identified in its physiological role that perhaps may or may not contribute to renal status and quality of life through. [25]

Despite these limitations, our study demonstrated the association between serum 25(OH)D and HRQOL in pre-dialysis CKD patients. Given that the serum 25(OH)D level can be easily identified in clinical practice and that treatment of vitamin D deficiency is simple and inexpensive, these results should be taken into consideration by clinicians in order to improve patient outcomes.

In conclusion, there was an independent and significant association between serum 25(OH)D levels and the KDCS and MCS score in pre-dialysis CKD patients, but not between vitamin D levels and PCS score.

## Supporting information

S1 File Raw data on KNOW-CKD patients in this study. (XLSX)
